# Protocol of the Luebeck longitudinal investigation of SARS-CoV-2 infection (ELISA) study – a prospective population-based cohort study

**DOI:** 10.1186/s12889-022-13666-z

**Published:** 2022-07-07

**Authors:** Alexander Balck, Bandik Föh, Max Borsche, Johann Rahmöller, Eva-Juliane Vollstedt, Frederike Waldeck, Nadja Käding, Christoph Twesten, Alexander Mischnik, Gabriele Gillessen-Kaesbach, Marc Ehlers, Christian Sina, Stefan Taube, Hauke Busch, Jan Rupp, Alexander Katalinic, Christine Klein

**Affiliations:** 1 Institute of Neurogenetics, University of Lübeck and University Hospital Schleswig-Holstein, Lübeck, Germany; 2 Department of Neurology, University of Lübeck and University Hospital Schleswig-Holstein, Lübeck, Germany; 3 Institute of Nutritional Medicine, University of Lübeck, Lübeck, Germany; 4 Department of Medicine I, University Hospital Schleswig-Holstein, Lübeck, Germany; 5 Department of Anesthesiology and Intensive Care, University of Lübeck and University Hospital Schleswig-Holstein, Lübeck, Germany; 6 Department of Infectious Diseases and Microbiology, University of Lübeck and University Hospital Schleswig-Holstein, Campus Lübeck, Lübeck, Germany; 7 Perfood GmbH, Lübeck, Germany; 8 Health Protection Authority, Lübeck, Germany; 9 University of Lübeck, Lübeck, Germany; 10 Institute of Virology and Cell Biology, University of Lübeck, Lübeck, Germany; 11 Lübeck Institute of Experimental Dermatology (LIED), University of Lübeck, Lübeck, Germany; 12 Institute of Social Medicine and Epidemiology, University of Lübeck, Lübeck, Germany

**Keywords:** Coronavirus, COVID-19, Pandemic, Surveillance, ELISA, Risk behaviors, Risk factors, Antibodies, Cohort-study

## Abstract

**Background:**

Considering the insufficiently controlled spread of new SARS-CoV-2 variants, partially low vaccination rates, and increased risk of a post-COVID syndrome, well-functioning, targeted intervention measures at local and national levels are urgently needed to contain the SARS-CoV-2 pandemic. Surveillance concepts (cross-sectional, cohorts, clusters) need to be carefully selected to monitor and assess incidence and prevalence at the population level. A critical methodological gap for identifying specific risks/dynamics for SARS-Cov-2 transmission and post-COVID-19-syndrome includes repetitive testing for past or present infection of a defined cohort with simultaneous assessment of symptoms, behavior, risk, and protective factors, as well as quality of life.

**Methods:**

The ELISA-Study is a longitudinal, prospective surveillance study with a cohort approach launched in Luebeck in April 2020. The first part comprised regular PCR testing, antibody measurements, and a recurrent App-based questionnaire for a population-based cohort of 3000 inhabitants of Luebeck. The follow-up study protocol includes self-testing for antibodies and PCR testing for a subset of the participants, focusing on studying immunity after vaccination and/or infection and post-COVID-19 symptoms.

**Discussion:**

The ELISA cohort and our follow-up study protocol will enable us to study the effects of a sharp increase of SARS-CoV-2 infections on seroprevalence of Anti-SARS-CoV-2 antibodies, post-COVID-19-symptoms, and possible medical, occupational, and behavioral risk factors. We will be able to monitor the pandemic continuously and discover potential sequelae of an infection long-term. Further examinations can be readily set up on an ad-hoc basis in the future. Our study protocol can be adapted to other regions and settings and is transferable to other infectious diseases.

**Trial registration:**

DRKS.de, German Clinical Trials Register (DRKS), Identifier: DRKS00023418, Registered on 28 October 2020.

**Supplementary Information:**

The online version contains supplementary material available at 10.1186/s12889-022-13666-z.

## Background

From initial local reports from China on infections with SARS-CoV-2 in December 2019, the disease rapidly evolved from an epidemic to a pandemic, with over 270 million confirmed cases and 5 million COVID-19 patients deceased worldwide [1]. The emergence of the new SARS-CoV-2 variant B.1.617.2 (Delta) in December 2020 and especially variant B.1.1.529 (Omicron) in November 2021^2^ led to vastly increased infection rates, including a significant number of vaccination breakthroughs [2]. While currently available vaccines against SARS-CoV-2 reduce the frequencies of severe cases and hospitalization rates with mostly mild cases in fully vaccinated patients, the impact of highly contagious variants such as B.1.1.529 (Omicron) is concerning because vaccination only has a limited effect on spread and development of COVID-19 [3, 4]. Thus, the emergence of new variants such as B.1.1.529 (Omicron) can quickly transform previous low-incidence areas into hotspots. In the municipal area of Luebeck in Northern Germany, where the current study protocol was initiated, the 7-day incidence rate rose from 20 infections per 100,000 inhabitants in October 2021 to 1190 infections per 100,000 inhabitants in February 2022 [5].

Considering the insufficiently controlled spread of new SARS-CoV-2 variants, low vaccination rates, and increased risk of post-COVID syndrome, well-functioning, targeted intervention measures at local and national levels are urgently needed to control the spread of SARS-CoV-2. Surveillance strategies should be as adaptable as possible to consider regional conditions and different spread scenarios. Surveillance concepts (cross-sectional, cohorts, clusters) need to be carefully chosen to monitor and assess incidence and prevalence at the population level to contain the massive spread of infections. Longitudinal studies in defined, population-based cohorts at the population level are currently scarce, often cross-sectional, and usually cover only relatively short time intervals [6]. Likewise, recently conducted antibody prevalence studies were conducted cross-sectionally. They covered only short periods in specific regions, such as different parts of Germany [7–12], Great Britain [13], northeastern Italy [14], California [15], Pakistan [16], Portugal, [17] and Peru [18].

A critical methodological gap for identifying specific risks/dynamics for transmission and post-COVID-19-syndrome includes repetitive testing of a defined cohort with simultaneous assessment of symptoms, behavior, risk, and protective factors, as well as quality of life. Therefore, a longitudinal, prospective surveillance study with a cohort approach was started in Luebeck in April 2020. The study was performed on people living in the City of Luebeck/Germany (approx. 220,000 inhabitants, population density approx. 1000/sqm). In the first part of the study, more than 3000 people, corresponding to approximately 1% of the catchment area in and around Luebeck, were examined in person at regular intervals. The results of this part of the study were recently published [19].

Methodological details on study design and protocol have not been previously reported and are presented here as an easily adaptable template for further studies, particularly in other regions. Furthermore, we present the protocol of a follow-up study, which will investigate the cohort after the sharp increase of cases in March 2022 due to the new SARS-CoV-2 B.1.1.529 (Omicron) variant [20]. Key focus of this follow-up study will be studying immunity after vaccination and/or infection, as well as post-COVID-19-symptoms in our defined cohort. For this, we have adapted our methods so that a study test center is no longer necessary and that participation can now be easily conducted from home.

In the following, we describe the study protocol and provide an easily transferable model for effective surveillance that allows for monitoring incidence prevalence, as well as re-infection rates, and enhancing preparedness for future potential pandemics. The study is funded by the National Research Network of University Medicine on COVID-19 in surveillance and testing (B-Fast work package 4), the Federal State of Schleswig-Holstein, and a crowdfunding campaign of the University of Luebeck.

## Methods

### Study aims

1. To monitor the prevalence and incidence of SARS-CoV-2 in the general population of the Luebeck municipal area
2. To estimate the extent of undetected SARS-CoV-2 infections in the general population of the Luebeck municipal area
3. To assess the influence of hygiene measures (e.g., mandatory mask-wearing) over time and in response to the implementation and release of official lockdown and containment measures.
4. To identify behavioral and occupational factors (e.g., occupation, working from home, using public transportation, meeting with others) that may increase the risk of infection and post-COVID-19-syndrome.

### Study centers

- Design and implementation of a local test center that meets pandemic hygiene regulations to perform recurrent nasopharyngeal swabs and blood collection for antibody testing in a cohort representative of the general population.
- Design and implementation of a data center to collect, assemble, and analyze pseudonymized study data from different sources (app, test center, diagnostic laboratories).

### IT infrastructure

- Study App (Perfood GmbH, Lübeck)
  - ◦ Cell-phone app for paperless, repeated application of questionnaires for the collection of standardized behavioral data
  - ◦ Questionnaire for follow-up testing
- Online Booking (www.timify.com), (Timify, München)
  - ◦ Booking timeslots for test center visits
- Subject database (In-house, web-based, MySQL, and PHP)
  - ◦ Contactless check-in at the test center
  - ◦ Documentation of test center visits
  - ◦ Pseudonymized sample assignment
- Sample database (In-house, web-based, MySQL, and PHP)
  - ◦ Tracking biological material at different locations (i.e., test center, diagnostic laboratories, biobank, data center)
  - ◦ Biobanking

### Setting

With approximately 220,000 inhabitants, the Luebeck area is part of the greater Hamburg metropolitan region and a major tourist destination in Germany. Around 7000 people commute between Luebeck and Hamburg, and an average of 50,000 tourists visit Luebeck per day [21].

### Study design

The study is designed as a prospective, longitudinal, surveillance cohort study, involving seven rounds of in-person testing with a representative and a risk-based sample of citizens of the Luebeck metropolitan area (total *n* = 3000) in a first part [19]. The second part involves a mostly self-reported follow-up on re-infections and current health status. The ethics committee approved both parts of the study protocol of the University of Luebeck. Study details were submitted to the German Clinical Trial Register (DRKS) (Identifier: DRKS00023418).

### Study procedures

#### Recruitment and sample

Initial cohort recruitment took place in April 2020 via local press and radio announcements, posters, flyers, an announcement by the mayor of Luebeck on the city’s official homepage, as well as University internal e-mail distribution. Inclusion criteria comprised residence in the metropolitan area of Luebeck, age 18 years or older, active consent for study participation, registration for study participation via the study app, and willingness and ability to come to the local study test center for regular PCR testing and antibody measurements. Study registration and consent were app-based, using an existing cell phone app (MillionFriends, Perfood GmbH) established by a start-up company that emerged from the University of Luebeck (see below).

Activation of the study app marked the start of the study and, based on the information provided in the initial questionnaire (e.g., age, occupation, gender), a sample representative of the Luebeck population (*n* = 2145) was drawn from 7303 registered individuals, as well as a risk group consisting of individuals with a high number of professional contacts with other people (*n* = 1374). Ultimately, 3474 individuals were selected and invited via an e-mailed link to make the first appointment at the study test center using an online appointment booking app.

#### App

An existing collaboration with Perfood GmbH (perfood.de) was utilized, and the study questionnaires were integrated into their existing app. No personal data other than an email address had to be entered in the app to participate in the study. A unique hash was generated by the app for each individual participant and used to link the app data to the study data in the data center. The QR-code of this hash was used for contact-less check-in at the study center. An initial questionnaire was started when logging into the app; after that, a follow-up questionnaire was activated every 3 days. Data was collected on the app data server and was accessible by the study’s data center.

#### Test center and initial testing procedure

In collaboration with the local health authorities, we established the test center at a local conference center (Media Docks Luebeck, https://www.mediadocks.de/), which was identified as a suitable study center due to various characteristics: (i) The central location in Luebeck’s city center and availability of parking, (ii) its layout with a total of six rooms that were 25–50 m^2^ in size that could all be accessed from the outside, thereby providing maximum safety at times of a pandemic; (iii) sufficient storage space and office space for administrative activities. Participants were invited by the study data center per email using the email address entered into the study app. The invitation also included links to the study homepage (https://elisa-luebeck.de), where participants could find the study information, consent documents, and a video explaining the examination procedure. Subjects were asked to make their bookings in advance using the online tool Timify (https://web.timify.com/), which limited appointments to five per 15 minutes to prevent clustering of subjects at the study center at any time. A call center gave support to participants each workday from 8 to 12 am. The check-in room was set up with two separate counters facing outwards. Subjects were able to check in using their personal QR code from the study app and a barcode-scanner linked to the subject database.

At the first study visit, information about the study, written informed consent for participation, and answers to any questions were provided by the medical staff at this counter. After consenting to the study, personal data (name, address, telephone number) were recorded in the subject database of the test center (Table 1). Uniquely generated 8-digit barcodes were placed on all collected biological materials allowing the unambiguous assignment of the samples to the respective study subject by the study data center but not the diagnostic laboratories. This barcode was also stored as the identifier for the sample database of the biobank. By having the participants take their own barcoded specimen tubes into their study rooms themselves, the risk of specimen mix-up was minimized. Subjects were assigned to a specific study room, where specially trained medical students or staff performed the swab and blood sample collection. After sample collection, participants exited the building directly from the study room. The first appointment took approximately 15 min per person, including check-in, obtaining informed consent, personal data collection, and sample collection. Subsequent appointments usually took less than 10 min, including just the check-in and sample collection.

**Table 1:** Collected proband data

Personal data	Questionnaire topics	Laboratory data
Name	Covid Symptoms	**In person visits**
Address	Daily activities	Nasopharyngeal swab for PCR for SARS-CoV-2
Email	Recent travel	EDTA blood for DNA analyses
Telephone number	Adherence to lockdown measures	Serum for antibody analyses
Sex	Allergies	**Follow-up**
Age	Medication and supplements	Dried blood spot for antibody analyses
Education	Medical history	Nasopharyngeal swab for PCR testing of respiratory viruses
Occupation	Smoking	
Weight	Alcohol consumption	
Height	Pets	
	Well – being	
	Vaccination status	

Once samples were collected, they were immediately stored at 4 °C until courier pick up at the latest at 8 am the following day.

The study center was operated in a two-shift system, where shifts overlapped for 30 minutes mid-day to avoid interruptions concerning sample collection. Shifts always started 30 minutes before the center opened to prepare check-in and study rooms. Depending on the utilization of the test center facilities, which could be estimated in advance based on the status of the online bookings, 4–6 medical students were assigned to each shift. Their main task was to collect samples, but they also provided organizational support, e.g., check-in of the test persons and sample management. A physician was always present at the study site to provide overall supervision of the study site, supervise blood and swab collection, and answer questions from both student personnel and participants at all times. During the initial visit phase, up to three physicians were simultaneously present at the study center. Testing was conducted from Monday to Friday from 8 am to 6 pm, and 9 am to 1 pm on Saturdays. On average, around 180 participants visited the study center per day, with a maximum of 230 and a minimum of about 50 participants.

**Fig. 1:**
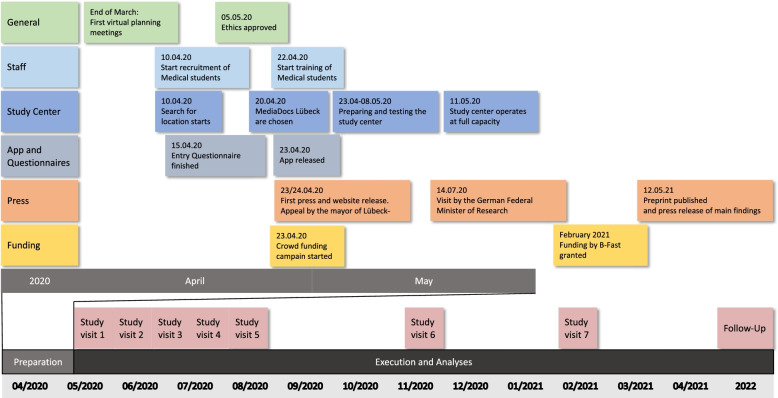
Timeline of the ELISA Study

Testing of the study cohort took place over 12 weeks from May to July 2020 at three-week intervals and in August 2020, November 2020, and February 2021 (Fig. 1). This period included important pandemic-related milestones, such as the end of the first lockdown, the summer holiday season with a high influx of tourists, the steep increase in infection rates in the fall/winter of 2020/2021, and the second lockdown.

For the second part of the study, participants will be invited via mail or email for follow-up testing on time point 8 and provided with a modified online questionnaire and instructions and material to prepare dried blood spots (DBS). These will be mailed back to the study center in a prepared envelope, where laboratory and statistical analyses will be conducted (s. below). In addition, we will ask 500 subjects to return to the study center to be tested via PCR regarding other respiratory viruses (s. below).

#### Reporting of findings

In case of positive PCR results, patients were immediately contacted by an assigned study physician, usually within 48 hours of sample acquisition, and local Health Authorities were notified. Depending on current regulations, patients were then quarantined between 10 and 14 days. Negative test results were sent to the participant within approximately 7 days by email in the study’s first phase. Antibody results were initially reported to the study participants by the study staff at the test center. Later, a personalized study portal was implemented, summarizing all study visits, including PCR and antibody tests results.

In the second phase of the study, participants without detectable SARS-CoV-2 antibodies will be informed after analysis of the data.

#### Personnel

The study involved a leadership team comprising seven specialists on clinical study design, epidemiology, diagnostics of infectious diseases, virology, immunology, software & database development, and ethics. A team of three full-time clinician-scientists was involved in the design, logistics, setting up of the test center, as well as training and supervision of the medical students. The test center was supported by 30 medical students and one secretary for the call center. PCR diagnostic teams were located at the University Hospital Schleswig Holstein (UKSH) and the Research Center Borstel. They involved a supervising diagnostician, as well as a team of at least two technicians or specifically trained life-science students on each location. PCR diagnostics were further supported by external diagnostic laboratories, including the LADR laboratory in Geesthacht (Hamburg) and Centogene (Rostock). The study further involved one computer scientist for data handling, study portal, and the online questionnaire and a courier driver for delivering the diagnostic samples.

#### Funding

Due to the rapid development of the pandemic, it was not possible to delay the start of the study until funding from the federal government became available. Governmental funds were not available until January 2021, 7 months after the beginning of the study. Until then, funds were raised by an extensive crowdfunding campaign orchestrated by the University of Luebeck that was announced in the local newspaper and on the university’s website. Local businesses, nonprofit associations, and residents donated more than 1,000,000€. In addition, the Federal State of Schleswig-Holstein provided 360,000€. The Possehl-Foundation will fund the second part of the study with 80,000€.

### Study instruments

#### Questionnaires

During the first part of the study, participants answered a baseline questionnaire and a recurring questionnaire stored in the study app every 3 days until September 2020, corresponding to study time points 1–5, and only once for time points 6 and 7 in December 2020 and February 2021 [19].

For the second part of the study, access codes for an online follow-up questionnaire will be sent to all participants via mail during March 2022 (Supplementary Material Q1).

This questionnaire will include symptoms of infection and post-COVID-19 syndrome, activities of daily living (such as shopping or attending events), information on adherence to lockdown measures, and related precautions (social distancing, home office, personal protective measures, limited mobility), contact with children, and self-reported results of previous SARS-CoV-2 testing (since the onset of the pandemic) (Table 1).

#### Laboratory analyses

In the first part of the study, we conducted diagnostics regarding acute SARS-CoV-2 infection with real-time quantitative PCR and SARS-CoV-2 Antibody diagnostics with ELISA as described in our previous publication (Table 1) [19, 22].

Serological tests of the second part of the study will be based on dried blood spots (DBS) as sample material for the follow-up testing (Table 1). The EUROIMMUN blood collection kit (ZV 9701–0101) allows convenient sampling at home. Capillary blood will be self-collected from the fingertip and applied onto blood collection cards. The five circles on the card have to be filled as completely as possible, and the blood droplets are left at room temperature for 3–4 h to dry. Afterwards, the card will be shipped to our laboratory for analysis. DBS will be punched out of the blood collection card for analysis, and the sample is then extracted from the DBS. The ELISA will be performed as previously described [19].

The second part also involves testing for other respiratory viruses (Table 1). Trained staff will take a nasopharyngeal swab (eSwabTM, COPAN) from a subset of 500 participants that will be invited to come to the study center. Swabs will be processed for nucleic acid extraction using the NucliSens® easyMAG™ platform (Biomérieux), according to the manufacturers’ instructions. Nucleic acids will be eluted, and quantitative RT-PCR is performed according to the manufacturers’ instructions using the *ampli*Cube Respiratory Viral Panel 1, 3, 4 and the *ampli*Cube Coronavirus Panel Kits (MIKROGEN, Neuried). Respiratory viruses include influenza A/B, SARS-CoV-2, MERS-CoV, hCoV (229E, HKU1, NL63, OC43), parechovirus, RSV, metapneumovirus, rhinovirus, enterovirus, and adenovirus.

### Statistics

#### Sample size calculation

No formal sample size calculation was made. With the resources available, it seemed possible to examine approximately 3000 individuals. We aimed for at least 1500 population-based participants and 1500 persons at risk, allowing overlap between both groups.

When planning the study, an official COVID-19 prevalence of 120/100,000 inhabitants was reported for the study region (Robert Koch Institute, April 27th, 2020). The true prevalence was assumed to be 5-fold higher (600/100,000) [23]. Based on a 1500 person sample a population-based infection prevalence rate of 0.60% with a 95% confidence interval of 0.31–1.05% could be determined. The precision of such an estimate seemed appropriate to us.

#### Data management

The subject and pseudonymized sample data are stored in MySQL databases self-hosted by the University of Luebeck. Access to the database is facilitated by a database web-frontend based on the CRUD database application dadabik™ developed by Eugenio Tacchini, also self-hosted at the University of Luebeck. An implemented user-rights management system allows for restricted and selective access to the study data. Participants were able to access selected data on a dedicated study website.

## Discussion

To combine and strengthen the activities of German University Medicine to cope with the current pandemic crisis, all 36 university hospitals joined forces nationwide to form the national research network “Network University Medicine” (NUM). The present study is included in the subsection *Nationwide Research Network Applied Surveillance and Testing (B-Fast)* in the population work package (https://www.umg.eu/forschung/corona-forschung/num/b-fast/). This work package tests the feasibility of new innovative surveillance concepts with selected sample testing in regional population clusters to develop best-practice models for surveillance of individuals infected with SARS-CoV-2 with and without symptoms.

Our study protocol allows for comprehensive population-based, prospective cohort surveillance. It also serves as a model for effectively monitoring the long-term dynamics of SARS-CoV-2 infection rates in fixed cohorts, identifying risk factors, and defining effective surveillance strategies. Due to its dynamic nature of mobility, the region of Luebeck is ideally suited as a model region for surveillance of SARS-CoV-2 infections. The used invasive methods are generally safe [24], and the app-based questionnaires make it possible to answer them regularly and remotely. Compared to face-to-face or telephone interviews used in other studies [11, 12], more interviews can be conducted due to the lower survey burden and lower costs. In addition, there is no risk of infection linked to in-person meetings. The test center was designed to minimize the time of stay, and no additional visit is necessary for the follow-up testing at time point 8.

Utilizing an existing app, medical students, and a discount for using the test center infrastructure further lowered the costs per proband. However, complete funding of the study was only secured 7 months after its beginning which posed a significant challenge for a timely study in a highly dynamic situation like a pandemic.

Our approach was well received by study participants, underlined by tight adherence to the questionnaires and very low dropout rates at follow-up testing during the study’s first phase. Another strength is the division of the cohort into both a representative sample of the local population and a group of individuals at greater risk of infection.

Limitations of the protocol can arise from its digital nature, as older individuals are less app-savvy or do not possess a smartphone. Participants were asked explicitly about problems answering the questionnaires during their first visit to the study center to mitigate these problems. A support hotline was set up to answer questions and was frequently used.

Initial study results have been published elsewhere and demonstrate vastly underestimated infection rates at the beginning of the pandemic [19]. The official trends of infection rates were well represented in our cohort. However, the absolute numbers were higher due to unrecorded cases. A high retention rate of 75–98% supports the feasibility of large-scale, long-term surveillance studies [19].

For our follow-up examination, we expect the seroprevalence to be remarkably higher than the preceding time points due to the more contagious variant B.1.1.529 (Omicron) and vastly increased infection rates worldwide, including our study area. With a current 7-day incidence rate of more than 1000 infections per 100,000 inhabitants in the municipal area of Luebeck, more than 1% of the overall population is being newly infected with SARS-CoV-2 every week. It needs to be emphasized that, although most cases in vaccinated patients have a mild disease course, there are still approximately 19% unvaccinated inhabitants above the age of four years in Germany at higher risk of severe courses [25]. In other developed countries like the USA or emerging countries, the rates of incompletely vaccinated people are even lower due to a lack of willingness or resources. Only 54% of the population worldwide have been vaccinated [26], which may even be an overestimate. Furthermore, high infection rates with SARS-CoV-2 are likely to aggravate long-term effects of the pandemic, particularly the prevalence of long- or post-COVID-19-syndrome. Although long-term effects occur more frequently in severe cases, up to 2.3% of mild COVID-19 cases show symptoms of post-COVID-19-syndrome more than 12 weeks after infection [27], highlighting the relevance of effective surveillance strategies even in a largely vaccinated population.

The ELISA cohort and our follow-up study protocol will enable us to study the effects of a sharp increase of SARS-CoV-2 infections on seroprevalence of Anti-SARS-CoV-2 antibodies post-COVID-19-symptoms and possible behavioral, occupational, and medical risk factors. We will be able to monitor the pandemic continuously and discover potential sequelae of an infection in the long term. Further examinations can be easily set up on an ad-hoc basis in the future.

Our study protocol can be adapted to other regions and settings and is easily transferable to other infectious diseases.

## Supplementary Information

**Additional file 1.**

## Data Availability

Not applicable.
