# Supplementary material for: Protocol of the Luebeck longitudinal investigation of SARS-CoV-2 infection (ELISA) study – a prospective population-based cohort study
Source: BMC Public Health. 2022 Jul 7;22:1305. doi: 10.1186/s12889-022-13666-z (PMC9261226; doi:10.1186/s12889-022-13666-z)
Supplement: Supplementary file 1 — Additional file 1. [file 12889_2022_13666_MOESM1_ESM.docx]

**Protocol of the Lübeck Longitudinal Investigation of SARS-CoV-2 Infection (ELISA) Study – a prospective population-based cohort study – Supplementary Material**

Alexander Balck, MD^1,2^, Bandik Föh, MD^3,4^, Max Borsche, MD^1,2^, Johann Rahmöller^3,5^, Eva-Juliane Vollstedt, MD^1^, Frederike Waldeck, MD^11^, Nadja Käding, PhD^11^, Christoph Twesten, MD^6^, Alexander Mischnik, MD^7^, Gabriele Gillessen-Kaesbach, MD^8^, Marc Ehlers,PhD^3^, Christian Sina, MD^3^, Stefan Taube, PhD^9^, Hauke Busch, MD^10^, Jan Rupp, MD^11^, Alexander Katalinic, MD^12^, and Christine Klein, MD^1*^

^1^Institute of Neurogenetics, University of Lübeck and University Hospital Schleswig-Holstein, Campus Lübeck, Lübeck, Germany

^2^Department of Neurology, University of Lübeck, Lübeck, Germany

^3^Institute of Nutritional Medicine, University of Lübeck, Lübeck, Germany

^4^Department of Medicine I, University Hospital Schleswig-Holstein, Lübeck, Germany

^5^Department of Anesthesiology and Intensive Care, University of Lübeck and University Medical Center of Schleswig-Holstein, Lübeck, Germany

^6^Perfood GmbH, Lübeck, Germany

^7^Health Protection Authority, Lübeck, Germany

^8^University of Lübeck, Lübeck, Germany

^9^Institute of Virology and Cell Biology, University of Lübeck, Lübeck, Germany

^10^Lübeck Institute of Experimental Dermatology (LIED), University of Lübeck, Lübeck, Germany

^11^Department of Infectious Diseases and Microbiology, University of Lübeck and University Hospital Schleswig-Holstein, Campus Lübeck, Lübeck, Germany

^12^Institute of Social Medicine and Epidemiology, University of Lübeck, Lübeck, Germany

*Correspondence to:

Prof. Christine Klein, MD

Institute of Neurogenetics

University of Lübeck and University Hospital Schleswig Holstein, Campus Lübeck

Ratzeburger Allee 160; D-23538 Lübeck

Phone: +49-451-3101-8200

E-Mail: christine.klein@neuro.uni-luebeck.de

**Supplementary Questionnaires**

Supplementary Questionnaire 1 - Initial Questionnaire

**General information:**

1. Please indicate your gender. (m/f/d)

2. Please indicate your year of birth.

3. Please enter your height.

4. Please enter your weight.

5. Please enter the zip code of the place where you currently spend most of your life.

**Current signs of illness / Signs of illness in the past:**

6. Which of the following signs of illness (symptoms) are you currently experiencing? (Multiple selections).

Fever / elevated temperature >37.5 °C

Cough

Do you have sputum?

What does the secretion look like? (multiple selection)

colorless/clear

whitish/mucilaginous

yellowish/greenish

bloody

Other (free text)

Shortness of breath

When does the shortness of breath occur (multiple choice)?

During exertion

At rest

When lying down

Other (free text)

Muscle/limb pain

Sore throat

Rhinitis

Is your nose blocked?

Do you have runny nose ("runny nose")

What does the secretion look like (multiple choice)

colorless/clear

whitish/mucilaginous

yellowish/greenish

bloody

Other (free text)

New onset of loss of smell/change in smelling

new onset of taste loss/change in tasting

Headache

Nausea

Vomiting

How many times have you vomited in the past 72 hours? (enter number)

Diarrhea

How many times did you have a bowel movement in the last 72 hours? (enter number)

Increased sweating

Chills

Seizures

Fatigue/increased tiredness

Other (free text)

I have no symptoms

6.1 Assuming you are currently suffering from signs of illness (symptoms), when did they start?

6.2 Apart from possible current signs of illness (symptoms), which of the following signs of illness were present in you between February 2020 and now? (multiple choice)

Fever / increased temperature >37.5 °C

Cough

Shortness of breath

Muscle / limb pain

Sore throat

Rhinitis

New onset loss of smell/change in smelling

New onset taste loss/change in tasting

Headache

Nausea

Vomiting

Diarrhea

Increased sweating

Chills

Seizures

Fatigue/increased tiredness

Other (free text)

I did not have any symptoms at any time

6.3. Assuming you had signs of illness (symptoms) between February 2020 and now, when did they first appear? (single choice)

First half of February

Second half of February

First half of March

Second half of March

First half of April

Second half of April

I had no symptoms at any time

**COVID-19: Contact and Testing:**

7. Have you had contact one or more times with a COVID19 patient(s) who tested positive?

Yes: When did the contact occur? (multiple choice)

First half of February

Second half of February

First half of March

Second half of March

First half of April

Second half of April

8. Have you been tested for coronaviruses?

Yes: 8.1 Have you been tested for coronaviruses one or more times? (single selection once/multiple times)

8.1.1 Testing once When was the testing done? (date; note to subjects: "If you are not sure, please select a date in the approximate time period!"))

8.1.2 Multiple testing

8.1.2.1 When was the first testing for Corona virus performed? (date selection; note to subjects: "If you are not sure, please select a date in the approximate time period!")

8.1.2.2 When was the last test for Corona virus (date selection; note to subjects: "If you are not sure, please select a date in the approximate time period!")

8.2 Was any of the tests positive?

**Hospitalization:**

9. Are you or have you been hospitalized since January 2020?

Yes: 9.1 When were you admitted to the hospital? (date selection, note to subjects: "If you are not sure, please select a date in the approximate time period!")

9.2 When were you discharged from the hospital? ("date selection" and "am currently still in the hospital," note to subjects: "If you are not sure, please select a date in the approximate time period!")

9.3 Is or was the hospitalization for a COVID-19 condition?

Yes: 9.3.1 Did you require oxygen during the stay?

9.3.2 Did you require ventilation in the interim?

**Travel:**

10. Have you been abroad between February 2020 and now?

Yes: 10.1 To which country did you travel? (multiple selection, if a country is clicked, window with question "when last?" and according to the month-half principle as in 7.)

Austria

Switzerland

France

Spain

Italy

USA

China

South Korea

Other (free text)

11. This question refers to travel within Germany: Have you been outside Schleswig-Holstein between February 2020 and now?

Yes: 11.1 Between January 2020 and now, have you been to (multiple choice, if a country is clicked, window with question "when last?" using the half-month principle as in 7.)

Hamburg

Berlin

Bavaria

Baden-Wuerttemberg

North Rhine-Westphalia

**Occupation and household:**

12. Indicate your highest school-leaving qualification (single choice)

Elementary school

Secondary school

Realschule

High school diploma

No school-leaving qualification

13. Have you graduated from university?

14. Do you belong to one of the following groups or do you work in one of the following professions?

Geriatric nurse

In a nursing home

Outpatient care service

Nurse in hospital

Physician in hospital

Other hospital staff (e.g. physiotherapist, dietician, cleaner)

Physician in private practice

General practitioner

Specialist

Pharmacist

Bus driver

Supermarket staff

Drugstore clerk

Seller at the weekly market

Letter or parcel carrier

Gas station employee

Hairdresser

Pupils/students

Teacher

Educator in day care center or kindergarten

Policeman/policewoman or employee of the public order office in field service

Member of the German armed forces/federal police force

Hotel employee (e.g. receptionist)

Service employee in the catering trade

Employee in delivery service

No, I do not belong to any of the listed groups

15. How many persons live in your household in total? (enter number)

16. How many people over the age of 60 live in your household in total? (enter number)

17. How many persons under 18 years of age live in your household in total? (enter number)

18. How many people in your household are employed in total? (enter number)

**Allergies:**

19. do you suffer from allergies in general?

Yes: 19.1 Which ones? (multiple choice)

Hay fever

House dust/mite

Animal dander

Medication

Perfume/cosmetics

Metals

Insect venom

Chemicals

Latex

Crustaceans/seafood

Cow's milk

Nuts/Peanuts

Sulfur dioxide and sulfites

Fruits/vegetables/grains (certain varieties)

Mustard

Sesame

Lupine

I have allergies, but I do not know which ones

Other (free text)

19.2 Do you currently suffer from allergy symptoms that you know of (watery eyes, runny nose?

**Pre-existing conditions and medications:**

20. Do you have one or more of the following pre-existing conditions? (multiple choice)

Asthma

Hypertension

COPD (chronic obstructive pulmonary disease)

Depression

Diabetes mellitus ("sugar disease")

Inflammatory bowel disease (Crohn's disease, ulcerative colitis)

Inflammatory diseases of the skin (e.g. psoriasis)

Elevated blood lipid levels

Fatty liver (NASH)

Condition after heart attack

Frequent urinary tract infections

Heart failure ( cardiac insufficiency)

Bone density reduction/osteoporosis

Cancer

Repeated sinus infections (sinusitis)

Condition after pneumonia

with viruses (e.g. influenza viruses)

with bacteria (e.g. pneumococci)

with fungi

do not know

Migraine

Parkinson's disease

multiple sclerosis

renal insufficiency (impaired kidney function )

Irritable bowel syndrome

Rheumatic disease of the joints

Rheumatic disease of internal organs/vessels

Hypothyroidism

Condition after stroke

Celiac disease

Other (free text)

I do not have any previous diseases

21. What medications do you take regularly? (multiple choice)

Antidiabetics (e.g. metformin, insulin)

Asthma inhalers (e.g., sultanol, budenoside)

Antihypertensives (e.g. metoprolol, enalapril)

Cortisone (e.g. prednisolone, hydrocortisone)

Immunosuppressants (e.g., azathioprine, methotrexate)

Antibodies/biologics (e.g. Humira, MabThera, Remicade)

Lipid-lowering agents (e.g. simvastatin, pravastatin)

Thyroid tablets (e.g. L-thyroxine, thyrostatic agents)

Painkillers: opiates (morphine, tramadol)

Painkillers: non-steroidal anti-inflammatory drugs (e.g. ASA, ibuprofen, diclofenac)

Other (free text)

I do not take any medication

22. Do you take dietary supplements (multiple choice)?

Vitamin D3

Vitamin C

Vitamin B1

Vitamin B3

Vitamin B12

Vitamin K

Vitamin E

Multivitamin preparation: Name (free text)

Magnesium

Zinc

Selenium

Silica

Other (free text)

I do not take any dietary supplements

23. Have you received one or more of the following vaccinations in your lifetime? (Multiple answer, click to open input year or "don't know")

Tuberculosis (BCG-TBC)

Yes: When last?

Pneumococcus ( pneumonia)

Yes: When last?

Influenza ( flu vaccination )

Yes: When last?

**Smoking and alcohol:**

24. Do you smoke? (single choice)

I have never smoked.

I smoke an average of 20 cigarettes or more a day.

I smoke an average of less than 20 cigarettes a day.

I once smoked an average of 20 cigarettes or more a day.

I once smoked an average of less than 20 cigarettes a day.

25. Do you drink alcohol? (yes/no)

Yes: How often? (single choice)

Daily

Several times a week

Once a week

Every two weeks

Rarely (e.g., only at parties)

25.1 If you drink alcohol, how many alcoholic drinks do you drink on average? (1 alcoholic drink = 1 beer (0.2l) or a glass of wine/champagne (0.1l) or 4cl spirits) (single choice)

1-2 alcoholic drinks

3-6 alcoholic drinks

7 or more alcoholic drinks

**Pets:**

26. Do you have pets?

Yes: 25.1 If yes, which ones? (multiple choice, if a question is clicked, the following question should appear: Has this pet currently or in the past few weeks shown signs of a cold "cold signs")?

Dog

Cat

Guinea pig

Rabbit

Bird

Fish/Amphibians/Reptiles

Other (free text)

Supplementary Questionnaire 2 - Recurring Questionnaire

Periodic questionnaire every 3 days.

**Current signs of illness:**

1. What signs (symptoms) of illness have you experienced in the last 72 hours? (Multiple selection, when selection is made, indented items will open).

Fever / increased temperature >37.5 °C

Cough

Do you have sputum?

What does the secretion look like? (multiple selection)

colorless/clear

whitish/mucilaginous

yellowish/greenish

bloody

Other (free text)

Shortness of breath

When does the shortness of breath occur? (multiple choice)

During exertion

At rest

When lying down

Other (free text)

Muscle/limb pain

Sore throat

Rhinitis

Is your nose blocked?

Do you have runny nose ("runny nose")

What does the secretion look like (multiple choice)

colorless/clear

whitish/mucilaginous

yellowish/greenish

bloody

Other (free text)

New onset of loss of smell/change in smelling

new onset of taste loss/change in tasting

Headache

Nausea

Vomiting

How many times have you vomited in the past 72 hours? (enter number)

Diarrhea

How many times did you have a bowel movement in the last 72 hours? (enter number)

Increased sweating

Chills

Seizures

Fatigue/increased tiredness

Other (free text)

I have no symptoms

2. Have you taken your body temperature in the last 72 hours?

Yes: What value did you take (enter; note to subjects: "If you took temperature more than once, please indicate the highest value.")

Where did you take your body temperature? (single choice)

Mouth

Armpit

Anus

Ear

Groin

3. Do you currently suffer from allergy symptoms you know (watery eyes, runny nose)?

**COVID-19: Contact and Testing:**

4. In the last 72 hours, have you had contact with one or more COVID19 patients who tested positive?

5. In the last 72 hours, have you had contact with anyone with flu symptoms (e.g., example of fever, cough, or sore throat)?

6. Have you been tested diagnostically, i.e., at a doctor's office, health department, or hospital, for coronavirus in the last 72 hours?

If yes: Was the test positive?

**Behavior:**

7. Have you been out in public in the last 72 hours?

Yes: Have you worn a mouth/nose guard in public in the last 72 hours?

8. Do you currently use another app to notify you of potential risk contacts related to COVID-19?

**Children, school, and daycare:**

9. are there children/youth under the age of 18 in your household?

Yes: 9.1 Have any of these children been to daycare/kindergarten in the last 72 hours?

kindergarten?

9.2 Were any of these children in elementary school in the last 72 hours?

9.3 Were any of these children in secondary school during the last 72 hours?

school?

**Work:**

10. did you leave your house to go to work in the last 72 hours?

Yes: 10.1 How many hours per day did you work away from home? (enter number)

10.2 Yes: How many colleagues did you have closer contact with (Less than 1.5 meters apart, more than 15 minutes) per work day? (single choice)

0-2

3-5

5-10

> 11

10.3 How many people outside your colleagues did you have close contact with in the work context per workday closer contact over a longer period of time (Less than 1.5 meters apart, more than 15 minutes)? (single choice)

0-2

3-5

5-10

> 11

10.4 How many people outside of your colleagues did you have close contact with in the work context per workday closer contact over a short period of time (Less than 1.5 meters apart, less than 15 minutes)? (single choice)

0-2

3-5

5-10

> 11

**Leisure:**

(Note to probands: Not all of the following questions need to be relevant at all times, but also refer to changes under a possible relaxation of the "lockdown.")

11. In the last 72 hours, have you been (multiple choice)

In a restaurant or coffee shop?

In a pub or bar?

At a discotheque or club?

At the movies, theater, or a concert?

in a sports hall, gym or similar?

at a doctor's office, physiotherapy or similar?

at a hairdresser, beauty salon or similar?

12. have you been shopping in the last 72 hours (multiple choice)

at the supermarket?

at the weekly market?

at the hardware store?

at the bakery?

at other retail stores?

13. in the last 72 hours, were you at an event with (Note to respondents: "If you were at more than one event, please select the one with the highest attendance." Multiple choice)

Less than 10 people

11 to 50 people

51 to 100 people

More than 100 people

Supplementary Questionnaire 3 – Follow-up

**General information:**

1. Please enter your year of birth.

2. Please enter your weight.

**COVID-19: Contact and testing:**

3. Have you had contact one or more times with a COVID19 patient(s) who tested positive? (Yes/No)

3.1. If yes, when did the contact occur? (Multiple choice)

1st quarter 2021

2nd quarter 2021

3rd quarter 2021

4th quarter 2021

1st quarter 2022

In the last 72 hours

4. In the past 72 hours, have you had contact with anyone suffering from flu-like symptoms (e.g., example of fever, cough, or sore throat)? (Yes/No)

5. Have you tested positive for Corona virus infection by antigen test (rapid test) one or more times? (Yes/No)

5.1. If yes, when was the first positive test?(Date; note to subjects: "If you are not sure, please select a date in the approximate time period."))

6. Have you been tested positive for Corona virus infection by PCR test once or more than once? (Yes/No)

6.1. If yes, when was the first positive test ? (Date; note to subjects: "If you are not sure, please select a date in the approximate time period."))

6.2 If you have had a history of COVID-19 disease, do you continue to suffer from symptoms that you attribute to the disease/that have persisted since the disease? (Yes/No)

6.2.1 If yes, which symptoms are present? (Multiple choice)

Fatigue

Headache

Breathing difficulties

Smell and taste disturbances

Impaired cognitive skills

Depressive mood

Sleep disorder

Anxiety

**Hospitalization:**

7. is there or has there been hospitalization for COVID-19 disease? (Yes/No)

7.1 If yes, did you require oxygen during your stay?

7.2 If yes, did you need to be ventilated?

**Household:**

8. How many people live in your household in total? (Enter number)

9. How many persons over 60 years of age live in your household in total? (Enter number)

10. How many persons under 18 years of age live in your household in total? (Enter number)

**Smoking and alcohol:**

11. Do you smoke? (Single choice)

I have never smoked.

I smoke an average of 20 cigarettes or more a day.

I smoke an average of less than 20 cigarettes a day.

I once smoked an average of 20 cigarettes or more a day.

I once smoked an average of less than 20 cigarettes a day.

12. Do you drink alcohol? (Yes/No)

12.1 If yes, how often? (Single choice)

Daily

Several times a week

Once a week

Every two weeks

Rarely (e.g., only at parties)

13. When you drink alcohol, how many alcoholic drinks do you drink on average? (1 alcoholic drink = 1 beer (0.2l) or a glass of wine/champagne (0.1l) or 4cl spirits) (single choice)

1 - 2 alcoholic beverages

3 - 6 alcoholic beverages

7 or more alcoholic beverages

**Pets:**

14. Do you have pets? (Yes/No)

**Current signs of illness:**

15. What signs (symptoms) of illness have you experienced in the last 72 hours? (Multiple choice).

Fever / increased temperature >37.5 °C

Cough

Shortness of breath

Muscle / limb pain

Sore throat

Rhinitis

New onset loss of smell/change in smelling

New onset taste loss/change in tasting

Headache

Nausea

Vomiting

Diarrhea

Increased sweating

Chills

Seizures

Fatigue/increased tiredness

Other (free text)

I have no symptoms

**Behavior:**

16. have you been out in public in the last 14 days? (Yes/No)

16.1. If yes: Have you worn a face mask in public in the last 14 days? (Yes/No)

17. Do you currently use an app that notifies you of potential risk contacts related to COVID-19? (Yes/No)

**Children, school, and daycare:**

18. Are there any children/adolescents under the age of 18 in your household? (Yes/No)

18.1 If yes: Have any of these children been to daycare/kindergarten in the last 14 days?

kindergarten? (Yes/No)

18.2. If yes: Have any of these children been to elementary school in the last 14 days? (Yes/No)

18.3 If yes: Have any of these children been to secondary school in the last 14 days? (Yes/No)

**Work:**

19. Did you leave your house to go to work in the past 14 days? (Yes/No)

20. In the last week, how many people did you have close contact with (less than 5 feet apart, more than 15 minutes)? (Single choice)

0 - 2

3 - 5

5 - 10

> 11

**Leisure:**

21. In the last 14 days, did you go to a (Multiple choice)

restaurant or café?

pub or bar?

discotheque or club?

cinema, theater or concert?

sports hall, gym or similar?

doctor's office, physiotherapy or similar?

hairdresser, beauty salon or similar?

22. Have you been to an event within the last 14 days (note to participants: "If you attended more than one event, please select the one with the highest attendance." Multiple choice)

Less than 10 people

11 - 50 people

51 - 100 persons

> 100 people

**Mobility:**

23. Did you use public transport in the last 14 days (Multiple choice)

Train

Bus

Train

Taxi/carpooling

Shared use of vehicles (e.g., "car sharing")?

**Psychological/medical aspects:**

24.Describe your current well-being on a scale of 1 - 10 (1 = low; 10 = high).

25. In the past 72 hours, how often have you been worried? (Scale 1-10; 1 = Not at all; 10 = Almost all the time).

26. How good was your sleep in the last 72 hours? (Scale 1-10; 1 = poorly; 10 = well)

27. How tired or exhausted did you feel in the last 72 hours? (Scale 1-10; 1 = Not at all exhausted; 10 = Very exhausted).

28 In the past 72 hours, how pronounced was your feeling of having symptoms of illness suggestive of infection with coronavirus? (Scale 1-10; 1 = Not at all; 10 = Very much).

29 In the past 72 hours, how involved did you feel socially? (Scale 1-10; 1 = Not at all involved; 10 = Very involved).

30. How often did you communicate via digital means (phone/video conferencing/chat) in the last 72 hours? (Scale 1-10; 1 = Not at all; 10 = Very much).

**Immunization:**

31. How many doses of vaccination have you received?

First vaccination dose (month/year) (Single choice)

BioNTech/Pfizer

Moderna

AstraZeneca

Johnson and Johnson

Novavax

Other (free text)

None

Second vaccination dose (month/year) (Single choice)

BioNTech/Pfizer

Moderna

AstraZeneca

Johnson and Johnson

Novavax

Other (free text)

None

Third vaccination dose (month/year) (Single choice)

BioNTech/Pfizer

Moderna

AstraZeneca

Johnson and Johnson

Novavax

Other (free text)

None

Fourth vaccination dose (month/year) (Single choice)

BioNTech/Pfizer

Moderna

AstraZeneca

Johnson and Johnson

Novavax

Other (free text)

None

32. When did you receive the last vaccination?

33. If I got the chance, I would get vaccinated (again, if necessary) (Single choice)

Definitely

Maybe

No

34. Have you received one or more of the following vaccinations in your lifetime? (Multiple choice, click to open input year or "don't know").

34.1 Pneumococcus (pneumonia)

If yes, when was the last time? (date)

34.2 Influenza (flu shot)

If yes, when was the last time? (date)
